# An aberrant precision account of autism

**DOI:** 10.3389/fnhum.2014.00302

**Published:** 2014-05-14

**Authors:** Rebecca P. Lawson, Geraint Rees, Karl J. Friston

**Affiliations:** ^1^Wellcome Trust Centre for Neuroimaging, University College LondonLondon, UK; ^2^Institute of Cognitive Neuroscience, University College LondonLondon, UK

**Keywords:** autism spectrum disorder (ASD), predictive coding, precision, sensory attenuation, learning, perception and action, sensory sensitivity, social interaction

## Abstract

Autism is a neurodevelopmental disorder characterized by problems with social-communication, restricted interests and repetitive behavior. A recent and thought-provoking article presented a normative explanation for the perceptual symptoms of autism in terms of a failure of Bayesian inference (Pellicano and Burr, 2012). In response, we suggested that when Bayesian inference is grounded in its neural instantiation—namely, predictive coding—many features of autistic perception can be attributed to aberrant precision (or beliefs about precision) within the context of hierarchical message passing in the brain (Friston et al., 2013). Here, we unpack the aberrant precision account of autism. Specifically, we consider how empirical findings—that speak directly or indirectly to neurobiological mechanisms—are consistent with the aberrant encoding of precision in autism; in particular, an imbalance of the precision ascribed to sensory evidence relative to prior beliefs.

## Introduction

The challenge of inferring the causes of our sensory inputs—arguably the goal of successful (optimal) perception—is twofold. Firstly, different causes can generate the same sensory input: the shadow that takes the form of a rabbit can be caused by a real rabbit or my hands configured to look like a rabbit. This inherent ambiguity induces something called an “inverse problem” for perception that can only be resolved in terms of prior beliefs about how our sensations are generated. The second problem is that it is not sufficient just to identify a plausible explanation for sensations, we also have to estimate the confidence we place in sensory evidence, relative to our prior beliefs. Disregarding, or failing to accurately estimate, confidence in sensory information—when making perceptual inference—would be like making a statistical inference without knowing the standard error of some estimated treatment effect. Just as statistical inference would be invalid if the standard error was not estimated properly; our perceptual inference would be sub-optimal if the estimation of confidence in sensory evidence, relative to prior beliefs, was compromised.

From a Bayesian perspective, our senses are bombarded with input from the world and our brain is constantly generating hypotheses about the causes of the sensory evidence it receives. These hypotheses can be regarded as prior expectations, which are generated over multiple timescales, from milliseconds to minutes, and over multiple levels of (hierarchical) description, from visual patterns to facial expressions. Predictive coding formulations of perception (e.g., Friston, 2005; Bastos et al., 2012) propose that expectations in higher brain areas generate top-down predictions that meet bottom-up stimulus-bound signals in lower hierarchical (sensory) areas. The discrepancy between the sensory input and descending predictions of that input is known as the prediction error. This prediction error reports what stimulus-associated information is “newsworthy” in the sense that it was unpredicted and informative. This information is passed up the hierarchy to inform higher-level expectations, which subsequently generate better predictions and thereby resolve prediction errors. The influence of (top-down) prior beliefs, relative to (bottom-up) sensory evidence, is controlled by the precision, or confidence placed in prediction errors at each level of the hierarchy (Friston, 2008). A high sensory precision will increase the influence of ascending prediction errors by turning up the “volume” of sensory channels in which we place more confidence. Conversely, a low sensory precision, relative to the precision of prediction errors higher in the hierarchy, will bias perception towards prior beliefs. Crucially, if the predictive coding account on offer is true, precision itself has to be estimated, much like estimating a standard error in statistics, in terms of its expectation. This leads to the notion of expected precision. In what follows, we will use precision to mean expected or subjective precision.

In predictive coding schemes, action and perception attempt to minimize prediction errors induced by sensory input at all levels of the hierarchy—to optimize posterior beliefs (expectations) about the causes of sensations, at multiple levels of abstraction. In this setting, perception is the process of minimizing prediction errors by providing better top-down predictions, while action minimizes prediction errors through a selective sampling of sensory input to ensure it conforms to our predictions. Crucially, both action and perception rest on an optimal representation of uncertainty; namely the precision of prediction errors at different hierarchical levels. This precision itself has to be estimated and deployed in an optimal way. Previously, we proposed that high level (prior) precision may be attenuated in autism, relative to sensory precision (Friston et al., 2013). In other words, in autism there may be a failure to attenuate sensory precision and contextualize sensory information in an optimal fashion. For example, an individual who always expects a high sensory precision would, to some extent, be a slave to their senses, affording sensations disproportionate weight in driving beliefs about their world. We will see below that, physiologically, optimizing precision corresponds to neuromodulatory gain control of neuronal populations reporting prediction error. This provides an important link between abnormalities of neuromodulation and false inference under predictive coding—a link we now pursue in the context of autism.

In what follows, we critically review neurobiological evidence consistent with an aberrant precision hypothesis. First, we will describe empirical studies that directly assess—or speak to—neurobiological correlates of prediction errors and their suppression in autism. Secondly, we will discuss the neuromodulatory gain control mechanisms that are implicated in encoding precision, and evidence pertaining to their disruption in autism. We finish by considering how the aberrant precision account of autism differs from other related recent accounts of autism. Although there is a large amount of evidence for predictive coding *per se*, the evidence for aberrant precision in autism (APA) is—at this stage—circumstantial and suggestive. Our main purpose is to motivate a process theory that equips functionalist or normative accounts with a biological mechanism and thereby highlight outstanding questions that, in principle, could be resolved using psychophysical and electrophysiological studies.

## Perception and action in autism

### Predictive coding in the brain

Our treatment will focus on the role of precision in predictive coding. Predictive coding is a neurobiologically plausible account of circumstantial physiological and anatomical evidence for predictive coding in the brain—ranging from formal or theoretical treatments (reviewed in Friston, 2008), through the anatomy of extrinsic connectivity and canonical microcircuits (reviewed in Bastos et al., 2012), to the functional architecture of the motor system (reviewed in Adams et al., 2013a). In this setting, precision is thought to be mediated by the gain or excitability of (superficial pyramidal) cells encoding prediction errors (Feldman and Friston, 2010; Shipp et al., 2013). We will adopt this theoretical perspective as a starting point for considering autism; acknowledging that there are many other interesting issues that can be addressed in terms of precision (for example attention, affordance, visual search, illusions etc., Feldman and Friston, 2010; Adams et al., 2013a,b; Brown et al., 2013). When appropriate, we will refer to the (computational and psychophysical) literature on precision to highlight the explicit connections with pathophysiology in autism.

### Sensory attenuation

Subjective or expected precision can play a key role in modulating the dynamics of perception. For example, if the precision of sensory evidence is increased, or if top-down prior precision is decreased, perception (posterior beliefs or expectations) will be dominated by sensory input. In other words, if you expect highly precise sensory input, you will increase the gain of sensory prediction errors at the expense of higher level expectations. Consequently, generalizable high-level causal structure will be sacrificed to accommodate overly accurate explanations of (potentially noisy) bottom-up sensory input. Autistic perception has previously been characterized by just such a lack of central coherence (Frith and Happé, 1994); specifically, a superior focus on the local aspects of a scene at the expense of the global “bigger picture” that is accompanied by an overwhelming sense of “sensory overload” (Simmons et al., 2009). These observations are supported by a plethora of behavioral data demonstrating impairments of individuals with autism in tasks that require integration of global attributes—such as global motion coherence (Milne et al., 2002; Pellicano et al., 2005), and superior performance on tasks that require identification of the individual parts of a visual stimulus—such as visual search (O’Riordan et al., 2001; O’riordan, 2004) and the embedded figures test (EFT; Frith and Happé, 1994; Happé, 1999; though see White and Saldaña, 2011).

A common finding, across a number of functional magnetic resonance imaging (fMRI) studies investigating the EFT in autism, is increased visual cortical activation, and decreased prefrontal activation in autistic participants relative to controls (Ring et al., 1999; Lee et al., 2007; Manjaly et al., 2007). Increased visual cortical activation in spatial-contextual processing tasks is typically interpreted as being consistent with increased bottom-up visual processing in autism. In the context of predictive coding, cortical responses are generally considered an index of (precision weighted) prediction errors (Friston, 2005). As such, these findings are consistent with the exuberant (stimulus-bound) production (and perceptual resolution) of sensory prediction errors, whose precision has not been appropriately attenuated.

A key tenet of this explanation is that high sensory precision in autism, relative to prior precision, may be caused by a failure of sensory attenuation. In other words, an inability to contextualize sensory information renders sensory prediction errors too precise and context insensitive. The effects of sensory precision can present in several guises. For example, repeating a stimulus generally leads to an adaptive attenuation of evoked neuronal responses. This is termed *repetition suppression* or *adaptation* in fMRI, and has been studied with the *mismatch negativity* (MMN) in electroencephalography (EEG). This effect can be understood as the suppression of prediction errors as the stimulus becomes more predictable (Summerfield et al., 2008; Ewbank et al., 2011). However, this repetition suppression can be reversed by attention (Kok et al., 2012)—an effect that can be attributed to an (attentional) increase in sensory precision (Feldman and Friston, 2010). Conversely, the exuberant responses associated with the MMN can, in part, the attributed to a failure to attenuate sensory precision during the processing of unexpected “oddball” stimuli (Garrido et al., 2009). Under conditions of high sensory precision, modeled and pharmacologically modulated MMN responses are diminished due to a failure to attenuate responses to repeated “standard” stimuli (Moran et al., 2013). Some MMN studies in autistic children also indicate diminished MMN amplitudes (Dunn et al., 2008) and latencies (Gomot et al., 2011) relative to neurotypical controls.

Though attempts to synthesize the MMN literature in autism reveal many inconsistencies (Marco et al., 2011), this may be due to differences in attentional demands used in different tasks (Dunn et al., 2008), which is consistent with the notion that attention is mediated by changes in expected precision during perception (Feldman and Friston, 2010; Vossel et al., 2013). Indeed, recent reports of larger extrastriate population receptive field maps (pRF) in autism (Schwarzkopf et al., 2014) echo the finding that pRFs increase under conditions of high attentional load (de Haas et al., 2014). This suggests that increased receptive fields in autistic subjects may reflect a failure to attenuate sensory precision.

One fMRI study has shown a failure to habituate to repeated presentations of the same faces in autism and, remarkably, the extent of this failure correlates with symptom severity (Kleinhans et al., 2009). These findings are consistent with a failure to suppress and contextualize prediction errors pertaining to the identity of faces over time. The consequence of this failure, especially for lower level sensory features, would be a constant state of sensory attentiveness, consistent with symptoms of sensory overload and oversensitivity to sensory stimulation in autism. The implication of these observations is that further studies of repetition (and expectation; Larsson and Smith, 2012; Todorovic and de Lange, 2012) suppression may be especially useful in establishing and quantifying a failure to contextualize or attenuate sensory precision in autism.

### Representing and responding to uncertainty

Another perceptual phenomenon that demonstrates the inferential nature of perception, and speaks to the balance of sensory drive relative to prior predictions, is binocular rivalry (Hohwy et al., 2008). When two different images are presented to each eye at the same time, one does not tend to perceive a fusion or mixture of the two images—as would be consistent with the (available) sensory evidence; instead, perception alternates between each monocular image as if trying to resolve the Bayesian inverse problem with prior beliefs about the causes of sensations. This Bayesian perspective is endorsed by studies showing that increasing the prior likelihood of one image can increase the duration that image is perceived relative to the competing image (Denison et al., 2011). Crucially, a recent binocular rivalry study of individuals with autism revealed increased durations of fused (mixed) percepts relative to controls—and also slower rates of perceptual alternation (Robertson et al., 2013; though see Said et al., 2013 for a negative result using simple stimuli). Robertson et al. note that simulations of binocular rivalry predict longer duration of mixed percepts by reducing neuronal inhibition or competition among high-level explanations for the sensory input. This is consistent with increased sensory precision, relative to the precision of prior beliefs that only one object can cause sensations at any one time (Hohwy et al., 2008).

Many visual illusions arise as a consequence of Bayes-optimal perception in artificially constructed circumstances. In the case of visual illusions, prior expectations generally reflect statistical regularities in the environment—and either arise from experience, such as the “light comes from above” prior (Adams et al., 2004) or may be hard-coded in the functional architecture of visual cortex. The illusory percept is “optimal” in that it is the best percept to explain the ambiguous sensory input on offer.

Interestingly, autistic individuals are reported to be less susceptible to a number of simple visual illusions (Happé, 1999). Additionally, a recent study shows that people with autism do experience the Sheppard illusion, although the magnitude is reduced (Mitchell et al., 2010) and autistic traits in a non-clinical sample have been shown to predict visual illusion magnitude for the rod-and-frame, Roelofs, Ponzo and Poggendorff illusions (Walter et al., 2009). Differences in the way visual illusions are administered and measured may account for contradictory results suggesting that people with autism experience visual illusions in the same way as neurotypicals (Mitchell and Ropar, 2004). Heterogeneity within the disorder (in precision dynamics controlled my neuromodulators—see below) may also account high variability in reported visual illusion magnitudes.

In neurotypicals, empirical and modeling studies of simultaneous luminance contrast illusions—under different levels of sensory precision—indicate that at very high levels of sensory precision, illusory percepts disappear (Brown and Friston, 2012). Although no studies have investigated these particular illusions in autism, the link between the precision of sensory evidence and the propensity to perceive illusory precepts offers an intriguing explanation for reports of reduced susceptibility to certain visual illusions in autism (and schizophrenia: e.g., Adams et al., 2013b).

Both rivalrous stimuli and visual illusions provide examples of perceptual uncertainty; circumstances where the available sensory evidence for a given perceptual “event” is ambiguous or imprecise. However, across time, uncertainty can also vary (this is called volatility) and adaptive behavior also rests on the accurate estimation of fluctuations in the precision (or inverse variability) of environmental contingencies. This estimation determines the weight one should place on sensory evidence relative to prior beliefs. Computational-fMRI studies have found trial-by-trial representations of environmental volatility in the anterior cingulate cortex (ACC; Behrens et al., 2007; den Ouden et al., 2010). Interestingly, structural (Haznedar et al., 1997) and functional (Lane et al., 1998) abnormalities in the ACC have been reported in autism and one fMRI study found increased activation in the ACC in autistic relative to neurotypical participants in a visual “oddball” detection task (Dichter et al., 2009). In this study, there were two types of oddball stimuli, “target” stimuli were infrequent but expected and “novel” stimuli were equally infrequent but unexpected. The authors found increased ACC response to targets, relative to novel stimuli, in the autism group. This could be interpreted as a failure to expect the unexpected—and further evidence for a failure to contextualize sensory processing in autism, in the face of uncertainty.

Finally, under the predictive coding framework, action changes sensory input in an attempt to minimize sensory (e.g., proprioceptive) prediction errors. This is usually cast in terms of classical motor reflexes involving the spinal-cord and cranial nerve nuclei (see Adams et al., 2013a; Shipp et al., 2013 for a review of the underlying functional anatomy). In this aspect of predictive coding, i.e., active inference, perception is entrained by action and sensory attenuation plays a key permissive role in action. Repetitive or stereotyped behaviors, clinically known as “stimming” (Wing, 1996), are characteristic of autism; these include behaviors such as hand flapping, head banging, and rocking back and forth. Within the predictive coding framework, stimming behaviors can be seen as attempts to create a sense of control via successful minimization of prediction errors through repetition of self-generated actions. In neurotypicals, there is a large body of evidence supporting the notion that the sensory consequences, and neural signatures, of self-generated actions are attenuated relative to externally generated actions—and a failure of this sensory attenuation in schizophrenia (Adams et al., 2013a; Shergill et al., 2014). In a state where all sensory inputs are in a sense unexpected, or associated with abnormally precise prediction errors, stimming, a predilection for sameness and a resistance to change become adaptive coping strategies—a way for individuals with autism to predict the sensory input they are receiving such that predictions or expectations are fulfilled in a consistent way. This is consistent with the (functionalist) view articulated by Pellicano and Burr (2012), but neurobiologically grounded in precision dynamics and synaptic gain control in the central nervous system (Adams et al., 2013b).

It is worth noting that, in addition to hypersensitivity to sensory stimulation, many people with autism can report hyposensitivities (Rogers and Ozonoff, 2005; Ben-Sasson et al., 2009). In addition to the coping strategies described above, withdrawal into oneself (American Psychiatric Association, 2013), can be seen as a means of avoiding the exuberant production of prediction errors. Such behavior resembles the “dark room problem” for predictive coding: if we are trying to minimize prediction errors, we might consider avoiding sensory stimulation and retire to a dark and quiet room (Friston et al., 2012b). An alternative explanation for withdrawal rests upon the failure to acquire internal models that are necessary for interaction with the world; particularly models of others that underlie prosocial exchange. This secondary consequence—of a primary failure to attenuate sensory precision—speaks to neurodevelopmental theories of autism. In summary, under predictive coding, the coping “atypical” behaviors produced by both hypersensitivity to sensory stimulation, and consequent withdrawal from affiliative and prosocial stimuli, can be seen as attempts to minimize the exuberant production of prediction errors resulting from imprecision in the balance of sensory evidence and top-down beliefs.

### Social interaction in autism: the greatest uncertainty?

So far, we have proposed that a single underlying pathology, aberrant encoding of precision, can explain the prominent features of autistic sensation, perception and action. Central to the theory of APA is the role of uncertainty. It is in uncertain situations that we rely most on our prior beliefs to contextualize and inform our perception. This means, one would expect the most marked deficits in perception and action in unpredictable situations that would normally call on precise prior beliefs. This provides a simple explanation for the pronounced social-communication difficulties in autism; given that other agents are arguably the most difficult things to predict. In the complex world of social interactions, the many-to-one mappings between causes and sensory input are dramatically increased and difficult to learn; especially if one cannot contextualize the prediction errors that drive that learning (Adams et al., 2013b).

Neuroimaging studies investigating contextual contributions to social interaction in autism are lacking. One fMRI study has demonstrated that neurotypical adults show a prediction error-like response in the superior temporal sulcus (STS) when eye gaze behavior violates expectations about where other people “ought to look”. Although autistic subjects show activity in the STS for dynamic shifts in eye gaze, they do not demonstrate this predictive effect (Pelphrey et al., 2005). This suggests a failure to predict gaze behavior in other people. Another fMRI study investigated how autistic children and neurotypical controls use contextual cues to interpret ironic verbal statements. They found group specific behavioral and neural differences: specifically, autistic children performed above chance but were less accurate than control children when relying on contextual information to interpret speech content. When interpreting the meaning of ambiguous utterances, the autistic group showed greater activation in inferior frontal and temporal brain regions (Wang et al., 2006). The authors argue this increased neural response reflects more effortful processing in the autism group, when trying to interpret the ambiguous meaning of utterances. However, increased activity in these regions could also be interpreted as an index of unsuppressed prediction errors, when content (the actual words spoken) and intended meaning (conveyed by contextual cues) could not be reconciled.

### Summary

Inferring the environment’s statistical structure and adapting behavior accordingly, is a fundamental problem that the brain appears to have solved. In the preceding section we reviewed empirical studies that directly assess—or speak to—neurobiological correlates of prediction errors and their suppression in autism. In so doing, we reviewed perception, action and social interaction during instances of high perceptual and environmental uncertainty and the, admittedly limited, evidence offered from existing studies is consistent with an imbalance of the precision ascribed to sensory evidence relative to prior beliefs. In particular, inappropriately high sensory precision may arise as a failure to attenuate sensory signals (i.e., a failure to contextualize sensory information in an optimal way) via top-down gain control. This, in turn, raises questions about perceptual learning and the acquisition of hierarchical models in the brain which we will return to later. Perhaps the most important inferences we make are about the intentions of others, which is clearly relevant for autism—and speaks to formal models of interpersonal inference as a promising avenue for autism research (e.g., Moutoussis et al., 2014).

## The neuromodulatory basis of precision

This section considers predictive coding as the neurobiological instantiation of Bayesian inference, where (expected) precision is mediated by the post-synaptic gain of superficial pyramidal cells encoding the prediction error (Feldman and Friston, 2010; Adams et al., 2013b; Shipp et al., 2013). The premise is that the perceptual and behavioral characteristics of autism can be considered as false inference about the causes of sensory input due to a failure attenuate sensory precision, relative to prior precision. Neurobiologically, this translates into a failure of (top-down) postsynaptic gain control of neuronal populations in the superficial cortical layers of sensory cortex. Post-synaptic gain gates the influence of presynaptic inputs on postsynaptic outputs and is determined by a number of factors. One key determinant of synaptic gain is the action of classical neuromodulators including acetylcholine, dopamine and serotonin. In what follows, we will briefly review the evidence for alterations in these neuromodulatory systems in autism, and how these alterations might relate to aberrant precision weighting in the disorder.

### Glutamate/GABA

It has been hypothesized for over a decade that autism might have a hypoglutamatergic basis: Abnormalities of the glutamate neurotransmitter system have been found in autism post mortem (Purcell et al., 2001) and a recent study showed that subcortical glutamate is reduced in autistic adults (Horder et al., 2013). Remarkably, baseline subcortical glutamate was found to correlate negatively with social communication impairments. In neurotypicals, visual γ-band (30–70 Hz) activity—that has been associated with the broadcasting of prediction errors (Arnal et al., 2011)—correlates with concurrently measured glutamate levels; demonstrating a functional role for glutamate in shaping the dynamics of visual cortical responses (Lally et al., 2014). In predictive coding, NMDA glutamate receptors (NMDAR’s) are hypothesized to play a role in gain control and contextual modulation (Friston, 2002, 2005). Hypoglutamatergic pathology in autism would therefore be consistent with context insensitive sensory drive and functional failures to optimize precision. In particular slow-acting voltage sensitive NMDAR’s are hypothesized to modulate feedback influences from higher-levels of processing onto lower levels (Friston, 2002, 2005) and so hypoglutamatergic pathology in autism would imply reduced prior precision in higher cortical areas. Indeed one possible mechanism that we offer for increased sensory precision is a failure of sensory attenuation (see above), which itself implies a reduced top-down influence on sensory drive.

While there are some important limitations on the applicability of mouse-models of cognition to human disorders, adult NR1(neo-/-) transgenic mice, which demonstrate NMDAR hypo-function, show abnormal behaviors consistent with autism symptoms, including: reduced social interactions, repetitive self-injurious behavior and sensory hypersensitivity. Gandal et al. (2012) recently investigated sensory processing in NR1(neo-/-) mice and found many sensory electrophysiological endophenotypes consistent with human studies of autism, including reduced pre-pulse inhibition (a measure of sensory gating) (Yuhas et al., 2011), reduced auditory-evoked latencies (Roberts et al., 2010) and reduced γ-band synchrony (Gandal et al., 2010). This encouraging mouse-model of autism may provide a preliminary link between hypo-glutamatergic pathology and the precision-based gating of ascending sensory signals (i.e., prediction errors).

### Acetylcholine

There is a large body of evidence linking acetylcholine to the encoding of perceptual and environmental uncertainty; in particular suggesting a role for acetylcholine in suppressing top-down influence on stimulus driven cortical responses (Yu and Dayan, 2005). Recently, optogenetic stimulation of the basal forebrain, the main source of cholinergic modulation in the cortex, has been shown to enhance V1 responses to visual inputs, desynchronise neural spiking and produce a behavioral improvement in tests of visual discrimination (Pinto et al., 2013). Conversely, basal forebrain inactivation depresses visual responses, synchronizes spiking and impairs discrimination performance—providing an elegant demonstration of how acetylcholine can modulate the (putative) precision of visual prediction errors. In the auditory domain, cholinesterase inhibition attenuates neural suppression in humans as a function of stimulus repetition (Moran et al., 2013). This is consistent with acetylcholine increasing the precision of sensory prediction errors, resulting in a failure to attenuate overall electrophysiological response with stimulus repetition.

In autism, reports of basal forebrain neuron pathology (Kemper and Bauman, 1998), morphological abnormalities (Riva et al., 2011) and reduced cortical cholinergic receptor function (Perry et al., 2001) are consistent with the idea of aberrant cholinergic encoding of precision in autism, and selective cholinergic interventions are considered a fruitful avenue for development of autism therapeutics (Deutsch et al., 2010). In another recent mouse model of autism, systematically increasing the availability of acetylcholine was found to alleviate behavioral symptoms consistent with autism (Karvat and Kimchi, 2013). While at first this might seem at odds with the suggestion of increased sensory precision in autism it is worth noting that the strain of mice used in this study express chronically low brain acetylcholine levels in the medial prefrontal cortex (McTighe et al., 2013). This might indicate that the behavioral effects reported in Karvat and Kimchi (2013) arise by restoring medial prefrontal acetylcholine availability; i.e., restoring precision dynamics at higher cortical levels such that sensory precision is no longer (relatively) high. However, while there are many reports of abnormal medial prefrontal cortex function in autism (Gilbert et al., 2008; Watanabe et al., 2012), there is, at present, little neurochemical evidence for low brain acetylcholine levels in human autism.

Furthermore, acetylcholine is not only involved in “attention-like” (Feldman and Friston, 2010) modulation of perception, but is also implicated in widespread facilitation of many cognitive processes, including novelty processing (Pepeu and Giovannini, 2004), conscious awareness and sleep states (Perry et al., 1999) and interacts with other monoaminergic neurotransmitter systems. In summary, acetylcholine may mediate the dynamics of precision in perceptual inference and may be an important candidate target for pathophysiology in autism. At this stage, however, more pharmacological and neuroimaging studies are necessary to better characterize the role of cholinergic neuromodulation in autism.

### Monoamines

Detailed investigations of the serotonin and dopamine systems in autism are sadly lacking and the studies that do exist are far from conclusive. Clinical observations suggest that dopamine-blocking antipsychotic drugs reduce repetitive and self-injurious behaviors in autism (Posey et al., 2008) and studies indicate that dopamine transporter binding is significantly higher in the orbitofrontal cortex of autistic adults relative to controls (Nakamura et al., 2010). In the context of active inference, dopamine is proposed to mediate the precision of cues (with affordance) that induce behavior (Friston et al., 2012a). Additionally, dopamine is converted to norepinephrine pre-synaptically via the action of dopamine β-hydroxylase (DBH). This means that dopamine and DBH availability are crucial determinants of norepinephrine function. Norepinephrine itself has a demonstrable role in encoding “unexpected uncertainty” in perceptual learning tasks (Yu and Dayan, 2005), and norepinephrine/DBH abnormalities in autism are some of the earliest (Lake et al., 1977) and most consistent neurobiological findings in people, and first-degree relatives (Robinson et al., 2001), with autism.

Other monoamines, such as serotonin have been implicated in the pathophysiology of autism (Harrington et al., 2013). Empirically, serotonin transporter binding is negatively correlated with dopamine transporter binding in the orbitofrontal cortex of people with autism and, across the whole brain, global serotonin transporter binding is reduced (Nakamura et al., 2010). This is suggestive of a role for serotonin/dopamine interactions in the neuromodulatory pathophysiology of autism. Common anti-depressant drugs, which block the reuptake of serotonin, are routinely prescribed to treat the core-symptoms of autism. However, a review of randomized control trials suggests there is limited evidence overall for the efficacy of serotonergic treatments (Kolevzon et al., 2006). Interestingly, serotonin has a neuromodulatory effect on cortical inhibition and excitation via interactions with glutamate and GABA (Ciranna, 2006), a potential route by which SSRIs could be efficacious in treating some individuals with autism, via gain control of cortical responses reporting prediction errors.

### Oxytocin

Oxytocin is a neuromodulatory hormone that plays a critical role in gestation and interacts with many of the neuromodulators outlined above (Richard et al., 1991). Plasma oxytocin levels are reduced in autistic children (Modahl et al., 1998) and intranasal oxytocin administration increases facial emotion recognition and amygdala responses to emotional faces in adults with Asperger syndrome (Domes et al., 2014). A recent study suggests that oxytocin increases the salience of socially meaningful stimuli, and attenuates the saliency of non-social stimuli, in children with autism (Gordon et al., 2013). This might suggest that oxytocin plays a role in contextualizing the precision of social and non-social cues. Mechanistically, this could be mediated via oxytocin-glutamate interactions that have been shown to modulate sensory-motor gating (Feifel and Reza, 1999). By implication, aberrant oxytocin function in autism may be manifest as a failure to contextualize and differentially attenuate multimodal cues—especially interoceptive cues either as a direct result of oxytocin dysfunction or as a result of subsequent failures of social learning.

### Summary

In the preceding section, we considered the putative neuromodulatory basis of how precision is mediated in the brain and evidence of abnormalities in these neuromodulatory systems in autism. The relative action and influence of these neurotransmitters may be largely related to timing, with NMDAR’s modulating the gain of sensory prediction errors, perhaps locally, over relatively short temporal contexts (in the order of ∼50 ms) (Friston, 2005) and neuromodulators like dopamine, acetylcholine and serotonin perhaps shaping the more enduring (contextual) aspects of precision in fronto-striatal and sensory systems (Corlett et al., 2009). To assert that the aberrant encoding of precision is the single underlying neuropathology in autism is a broad statement that is intended to acknowledge the myriad interacting neuromodulatory systems that may be involved.

In one sense, the potential explanatory potential of the aberrant precision proposal is that it accommodates a diversity of neuromodulatory mechanisms under one functional umbrella; namely the mediation of precision in hierarchical predictive coding. Furthermore, deficits in this singular function have the potential to explain a wide range of perceptual and behavioral abnormalities—of which a striking number are found in autism.

## Related accounts of autism

A recent influential proposal suggested that attenuated Bayesian priors might be responsible for the unique perceptual experiences in autism (Pellicano and Burr, 2012). Our commentary on that article suggested that under predictive coding, prior precision—encoded by neuromodulatory gain control mechanisms—may be attenuated in autism, relative to sensory precision (Friston et al., 2013). Related discussions also viewed predictive coding as a plausible framework to understand autistic perception (van Boxtel and Lu, 2013) and also suggested that individuals with autism might exhibit high precision at the sensory level (Brock, 2012; Van de Cruys et al., 2013) which may result in highly precise priors (Van de Cruys et al., 2013). Although, from the functional perspective, a decrease in prior precision results in the same posterior expectations as an increase in sensory precision; we have focused on the neurobiological mechanisms, suggesting that high sensory precision may arise as a failure to attenuate sensory precision and thereby contextualize sensory evidence in relation to prior beliefs.

Although mechanistically distinct, both overly precise estimates of sensory precision and under-precise estimates of prior precision would produce the same functional consequences; i.e., perception/interaction that lies closer to the sensory input and is insensitive to context. Here, we extend this notion to suggest that the sensory problems, repetitive and stereotyped behaviors and difficulties with social interaction in autism may all lie in the delicate balance of precision ascribed to sensory evidence relative to prior beliefs. Even if the primary deficit is expressed at the sensory level of cortical hierarchies, the secondary consequences of this, in terms of perceptual learning (and compensatory changes in neuromodulation at higher levels), necessarily requires one to consider the central role of hierarchical inference in perception and behavior. In this sense statistically “optimal” perception and learning go hand in hand and the relative *updating of precision* is just as important for sensation, perception and social interaction as the updating of predictions. See Mathys et al. (2011) for a formal illustration of this in the context of hierarchical Bayesian modeling of volatility.

Individual variability in basic physiological mechanisms, such as neuromodulatory function, may give rise to individual differences in learning and such differences may explain the heterogeneous nature of psychiatric diseases (Stephan et al., 2009). Indeed, some of the inherent heterogeneity of symptoms and behaviors within and between individuals with autism may be due to “state” problems with precision-weighting and the consequent (possibly compensatory) alterations to expected precision in higher levels of the cortical hierarchy. In support of this notion, recent study employing signal detection (SDT) measures found evidence in favor of attenuated priors in shaping perceptual inference in people with high levels of autistic traits (Skewes et al., 2014). In this case, the authors compared SDT measures that putatively map on to sensory precision and also prior beliefs and found it was the influence of prior beliefs that differed between high-and low autistic traits groups. In line with this finding, estimated individual differences in perceptual priors have been shown to predict perceptual performance in neurotypical adults (Stocker and Simoncelli, 2006). As such, individual differences (in precision-weighting) of learned expectations can shape individual differences in perception (Seriès and Seitz, 2013) and this is likely to vary between individuals across the autistic spectrum and also within the same individual. The implication of our formulation is that the normal variations in precision weighting become pathological in autism, or express themselves pathologically in situations where uncertainty is high.

We note that an aberrant precision account of autism—and associated normative accounts (Pellicano and Burr, 2012) accommodate many of the experimental findings that lead to existing theories of autistic behavior such as: a reduced sensitivity to context (Frith and Happé, 1994), general difficulties with predictability (Gomot and Wicker, 2012), difficulties with Theory of Mind (Baron-Cohen et al., 1985), reduced “top-down” control (Happé and Frith, 2006) and enhanced “bottom-up” functioning (Mottron et al., 2009). Anchoring Bayesian inference, specifically false inference, in predictive coding has the advantage of speaking to these cognitive, theoretical and normative accounts in a robust neurobiological and formal language (see Friston and Kiebel, 2009 for a detailed mathematical implementation of predictive coding, and Mathys et al., 2011 for learning under uncertainty).

### Summary

There are four key points that summarize the perspective offered by aberrant precision, in relation to its implicit normative account of autism. First, predictive coding and aberrant precision offer a neurobiological mechanism for the Bayesian formulation of Pellicano and Burr (2012). We have tried to substantiate this by drawing on computational and empirical evidence for an imbalance of sensory precision relative to top-down precision. Second, we have emphasized that the importance of sensory precision *relative* to the precision of prior beliefs, where an inaccurate estimate of either can, in principle, produce the same phenomenology. Specifically we outline an adaptive mechanism (a failure of sensory attenuation) that could underlie increased sensory precision. Third, we stress the importance of the dynamic evolution of precision at different temporal scales and levels of the cortical hierarchy (cf. Mathys et al., 2011). An important temporal scale here includes the learning of (relatively invariant) model parameters that renders the acquisition of generative models—particularly of self vs. others—sensitive to aberrant precision. Finally we highlight the fact that expected or subjective precision is not necessarily fixed and individual differences in the (tonic vs. phasic) availability of different neuromodulators can shape the perception and learning in both neurotypical and autistic people.

The debate between low “hypo-priors” (Pellicano and Burr, 2012) and decreased sensory noise (Brock, 2012) can be usefully contextualized under hierarchical predictive coding: the issue here is whether precision is too low at higher (prior) levels of the hierarchy, or too high at lower (sensory) levels. The original article by Pellicano and Burr did not refer to the mechanisms of predictive coding and offered a state (as if) account of autism. In contrast, predictive coding is a process theory that renders the distinction between aberrant precision at high and low hierarchical levels much more prescient. Functionally, we have noted that posterior expectations (although not posterior confidence) depend on, and only on, the relative hierarchical precision (e.g., Mathys et al., 2011). However, from a pathophysiological perspective, the hierarchical level of insult is clearly important. Although this issue is not easy to resolve given the current empirical evidence, our emphasis on a failure of sensory attenuation speaks more to Brock’s perspective than imprecise priors. Although the posterior expectation is unaffected by the relative precision, the posterior confidence will show opposite effects—with increasing posterior confidence under a failure to attenuate sensory precision and a decrease in posterior confidence with a loss of precise priors. Probing these predicted opposite effects will permit future empirical studies to begin to resolve this matter. Although this is an important issue, we have also emphasized that secondary changes in subjective precision (and perceptual learning) distant from the primary pathology may be an important etiological factor—particularly in a neurodevelopmental setting.

## Conclusions

Expectations about the precision of sensory inputs, relative to the precision of prior beliefs—encoded by neuromodulatory gain control mechanisms—may play a central role in coordinating the dynamics of perception, action, and social behavior. Here, we suggest that abnormalities in autistic perception, action and social interaction can be explained by an imbalance of the precision ascribed to sensory evidence relative to prior beliefs. We have attempted to demonstrate, with supportive empirical evidence, that the aberrant encoding of precision provides a parsimonious means of linking the sensory difficulties in autism to the pervasive social-communication problems. In particular, this account predicts the most pervasive difficulties in autism should emerge when environmental uncertainty is high, such as interpreting ambiguous or imprecise sensory input during visual illusions, rivalrous stimuli, and during social exchanges.

The aberrant precision account of autism has sufficient mechanistic and neurobiological specificity to generate testable and falsifiable hypotheses using clinical, behavioral, neuroimaging, pharmacological and computational modeling methods. While we acknowledge that the evidence for the aberrant precision account of autism is only suggestive at present, it is hoped that this principled functional and biologically grounded approach to psychopathology and pathophysiology of autism will generate new empirical studies, novel hypotheses and ultimately contribute to a better understanding of its neurobiological basis.

## Conflict of interest statement

The authors declare that the research was conducted in the absence of any commercial or financial relationships that could be construed as a potential conflict of interest.
